# Editorial: Physical Examination and Rehabilitation of Fasciae and Muscles in Sports Injuries

**DOI:** 10.3390/bioengineering12111238

**Published:** 2025-11-12

**Authors:** Carmelo Pirri, Nathaly Gaudreault

**Affiliations:** 1 Department of Neurosciences, Institute of Human Anatomy, University of Padova, 35121 Padova, Italy; 2 Centre de Recherche du CHUS, CIUSSSE de l’Estrie-CHUS, Sherbrooke, QC J1H 5N4, Canada; nathaly.gaudreault@usherbrooke.ca; 3 École de Réadaptation, Faculty of Medicine and Health Sciences, Université de Sherbrooke, Québec, QC J1H 5N4, Canada

## 1. Introduction

Sport injuries remain one of the most pervasive challenges in sport medicine, affecting athletes across disciplines and levels of performance. Despite advances in training methodologies, diagnostic imaging and rehabilitation technologies, soft tissue injuries continue to account for the majority of time lost from training and competition. For decades, the focus of both clinical evaluation and rehabilitation strategies has revolved around the structural and functional properties of muscle, tendons and joints. Yet in recent years, a paradigm shift has occurred. Increasing scientific attention has been directed toward the fascia, an extensive connective tissue network that not only envelops but also integrates and coordinates muscular and visceral function. Far from being an inert structural component, emerging evidence suggests that fascia may exhibit limited contractile behavior due to the presence of myofibroblasts and mechanosensitive cells. However, these properties and their physiological significance remain under investigation. Fascia is increasingly viewed as a sensory-rich structure potentially involved in proprioceptive and nociceptive modulation, although definitive functional evidence for these roles is still evolving. The fascial system’s multidimensional role, mechanical, neural and biochemical, demands a rethink of how clinicians assess, interpret and treat sports injuries. The traditional compartmentalized model, which isolates muscles from their fascial envelopes, has proven insufficient to explain phenomena such as persistent pain after apparent tissue healing, loss of flexibility without muscle lesion, or recurrent injury in spite of full strength recovery. The modern myofascial perspective offers a more integrative framework in which fascia and muscle are regarded as a single, functional unit. This viewpoint has profound implications not only for manual therapy and rehabilitation science but also for imaging, biomechanics and even bioengineering applications.

The Special Issue “*Physical Examination and Rehabilitation of Fasciae and Muscles in Sport Injuries*” brings together a series of studies that collectively advance our understanding of this integrated system. The included works span from fundamental reviews of fascial biology and pathology to clinical and translational studies employing advanced imaging, artificial intelligence and sensor technology. The articles illustrate the interplay between fascia and muscle across multiple levels, from microscopic mechanotransduction to macroscopic movement patterns and prosthetic signal transmission.

The first group of studies focuses on the physiological and pathophysiological aspects of fascia, examining its structure, adaptability and contribution to pain mechanisms. Kodama et al. [1] provide a detailed overview of fascial anatomy and functions, exploring how aging, hormonal status, repetitive mechanical load and inflammation shape fascial remodeling and stiffness. Cozacov et al. [2] extend this framework by presenting clinical evidence of differential sensitization between muscle and fascia in low back pain, emphasizing that both tissues play active, interacting roles in chronic myofascial pain syndromes.

A second group of articles investigates the mechanical and computational dimensions of the myofascial system, employing biomechanical modeling, wearable sensor data and artificial intelligence to analyze coordination variability and injury risk. Shao et al. [3] use deep neural networks to predict lower limb coordination during cutting maneuvers; Lin et al. [4] examine the kinematic adaptations of golfers with prior knee injury; and He et al. [5] review lower limb biomechanics in table tennis, highlighting the role of fascial continuity in energy transfer and performance optimization.

A final set of studies broadens the discussion to imaging and translational innovation. Pirri et al. [6] use ultrasound to assess the superior ankle retinaculum in football players with recurrent sprains, revealing fascial thickening and inhomogeneity as hallmarks of remodeling. Pogarasteanu et al. [7] explore the role of fascial tissue in electromyographic signal transmission, demonstrating how manipulation of fascial and subcutaneous layers can enhance myoelectric prosthesis control, thus linking fascial mechanics to bioelectrical communication.

Together, these papers articulate a coherent, multidisciplinary vision for the future of sport injury management, one that unites anatomy, physiology, biomechanics and engineering under the common denominator of the myofascial system.

## 2. Fascial Physiology, Mechanobiology and Pain Modulation

The review by Kodama et al. [1] lays the conceptual foundation for understanding fascia as a multifunctional tissue with unique mechanical and sensory properties. Unlike tendons or ligaments, which exhibit parallel collagen alignment, fascial collagen fibers are arranged in multiple orientations, allowing the tissue to accommodate multidirectional stresses. This architecture provides not only mechanical adaptability but also a substrate for complex force transmission between muscle groups. The authors emphasize that fascia contains abundant sensory nerve endings, including Ruffini endings and Pacinian corpuscles, making it one of the body’s richest sensory organs, these receptors contribute to proprioception, kinesthetic awareness and pain modulation, establishing fascia as a critical player in neuromuscular coordination.

Kodama and colleagues also describe how fascia dynamically responds to internal and external stimuli. Repetitive loading, such as that experienced during high-intensity training, can lead to fibroblast activation and transformation into contractile myofibroblasts, increasing tension and stiffness; conversely, disuse or hormonal changes can reduce collagen turnover, compromising elasticity. Aging is associated with dehydration and loss of hyaluronic acid viscosity, resulting in reduced fascial gliding and greater susceptibility to injury. The review highlights the pathological continuum between densification, reversible stiffening due to fluid loss, and fibrosis, irreversible structural alteration due to collagen crosslinking. Both processes can manifest clinically as restricted motion an chronic pain.

In a complimentary study, Cozacov et al. [3] examine the relationship between muscle and fascia in myofascial pain. Using ultrasound-guided needling of the quadratus lumborum, the authors assess pain responses in different tissue layers. Their results demonstrate that although fascia is hypersensitive in pain patients compared with healthy controls, muscle tissue generates more intense pain and more frequent twitch responses. This finding challenges the reductionist view that fascia alone is the main source of myofascial pain, instead suggesting a synergistic pathophysiology involving both tissues. The persistence of sensitization across acute and chronic conditions underscores the importance of early intervention and comprehensive treatment strategies that target the entire myofascial unit.

From a rehabilitation standpoint, these studies reinforce the need for multimodal approaches that combine manual fascial therapy, targeted strengthening and motor control retraining. They also highlight the potential of ultrasound and elastography to visualize fascial thickness, stiffness and hydration, offering clinicians quantifiable metrics for diagnosis and progress monitoring.

## 3. Biomechanics, Neural Control and Predictive Modeling in Sports

The mechanical behavior of fascia and muscle extends beyond static structure; it influences the dynamic coordination of movement. Shao et al. [3] integrate biomechanics and artificial intelligence (AI) to explore coordination variability of lower limb couplings during rapid directional changes, cutting maneuvers known to precipitate knee and ankle injuries. By applying long short-term memory neural networks to data from inertial measurement units, they achieve remarkable accuracy in predicting coordination patterns across different cutting angles. This methodological innovation allows movement variability, a known indicator of injury susceptibility, to be quantified and monitored outside laboratory environments. Such AI-driven models hold promise for real-time feedback systems capable of identifying hazardous movement patterns before injury occurs, bridging biomechanics with sport technology.

In parallel, Lin et al. [4] analyze the kinematics of golfers with a history of knee injury. Despite minimal differences in knee joint moments, injured players exhibit compensatory adaptations at the hip and ankle, including reduced hip flexion and greater ankle mobility during the downswing. These findings illustrate the principle of fascial continuity within the kinetic chain: when one segment is impaired, tension and motion redistribute across the myofascial network. Rehabilitation aims to restore functional integration across the entire kinetic chain, recognizing that fascial and muscular continuity underlies coordinated recovery. He et al. [5] contribute a systematic review examining lower limb biomechanics during the topspin forehand in table tennis. The synthesis of nineteen studies reveals that efficient stroke execution relies on a proximal-to-distal sequence of muscle activation, from the hip through the ankle to the wrist, mirroring the continuity of fascial lines described in anatomical studies. Hip rotation and ankle stability emerge as key determinants of performance, supporting the inclusion of lower-limb training and fascial flexibility work even in ostensibly upper body sports. The review also identifies knowledge gaps, such as the need for analysis of joint contact forces and coupling with racket–ball dynamics, which would further elucidate the mechanical contributions of the fascial system to complex athletic movements.

Collectively, these studies illustrate how fascial interconnections underpin the transfer of energy and coordination across the body. They also demonstrate that new computational tools and motion sensors can objectively capture these relationships, paving the way for personalized injury prevention and performance enhancement.

## 4. Fascial Imaging and Translational Bioengineering

Modern imaging and engineering approaches are transforming how fascial structures are assessed and manipulated. Pirri et al. [6] provide compelling evidence of fascial remodeling in the superior extensor ankle retinaculum (SEAR) of football players with recurrent sprains. Through ultrasound imaging, they document increased thickness and heterogenous echotexture on the injured side compared with both the contralateral and control ankles. These findings suggest that fascial tissues, much like ligaments, undergo structural adaptation after repeated trauma, potentially contributing to mechanical instability. The study highlights the diagnostic potential of ultrasound for fascial evaluation and recommends incorporating SEAR assessment into routine ankle examination, especially for athletes with chronic instability.

At the translational frontier, Pogarasteanu et al. [7] investigate how fascial tissue influences bioelectrical signal transmission in the context of myoelectric prosthesis control. Comparing healthy forearms and amputation stumps, they find that fascial and subcutaneous layers attenuate surface electromyographic signals. Remarkably, surgical reduction of these layers enhances signal strength and prosthesis responsiveness, demonstrating that fascial manipulation can optimize bioelectrical communication between muscle and device. This research extends the concept of fascial functionality beyond biomechanics, positioning fascia as an electrical modulator with implications for wearable sensors and rehabilitation technologies. The study bridges clinical anatomy and engineering, exemplifying how fascial insights can drive innovation in prosthetics and assistive devices, potentially influencing future sports performance monitoring systems. Together, these imaging and translational studies reinforce the idea that fascia is not only a mechanical structure but also a physiological interface between biological and technological systems. They demonstrate how quantifying fascial properties, such as thickness, stiffness and conductivity, can inform diagnosis, guide surgical or therapeutic interventions and inspire new bioengineering solutions.

## 5. Conclusions and Perspectives

The contributions to this Special Issue collectively affirm the central role of fascia in sports medicine and rehabilitation. Whether viewed through the lens of physiology, biomechanics, imaging or engineering, fascia emerges as an indispensable partner to muscle in determining movement efficiency, injury susceptibility and recovery potential. Several overarching principles can be distilled. First, fascia should be integrated into every stage of physical examination and rehabilitation. Palpation, ultrasound and elastography can reveal fascial restrictions or densifications that may not be apparent through muscle testing alone. Second, rehabilitation programs must adopt a myofascial approach, addressing both muscle contractility and fascial mobility to restore the natural balance of tension and elasticity. Third, advanced technologies, from wearable sensors to AI-based motion analysis, should be leveraged to capture fascial contributions to coordination and performance. Fourth, translational research linking fascial properties to bioelectrical signal quality opens new frontiers for prosthetic control and wearable rehabilitation devices.

Future research should aim to standardize fascial imaging protocols, develop validated clinical scales for fascial dysfunction and explore longitudinal changes in fascia during training, injury and recovery. Mechanistic studies at the cellular and molecular levels will elucidate how fibroblasts, extracellular matrix components and neural elements interact under athletic loading. Integrative models combining mechanical, neural and electrical aspects of fascia are needed to unify these insights. Ultimately, embracing the fascia–muscle continuum as a functional and diagnostic entity will enable more personalized, evidence-based interventions that enhance performance, accelerate recovery and reduce injury recurrence.

## Abbreviations

The following abbreviations are used in this manuscript:

AI	Artificial intelligence
SEAR	Superior extensor ankle retinaculum
